# Prognosis and Immunotherapy Effect of Triple-Negative Breast Cancer by Lactylation-Related Genes and Experimental Validation

**DOI:** 10.32604/or.2026.078051

**Published:** 2026-06-16

**Authors:** Yang Wang, Ying Xie, Yiyi Ye, Youyang Shi, Feifei Li, Mengdie Zhu, Ciyi Hua, Yuan Xu, Rui Yang, Sheng Liu

**Affiliations:** 1 Integrated Traditional Chinese and Western Medicine Breast Department, Longhua Hospital, Shanghai University of Traditional Chinese Medicine, Shanghai, China; 2 China Academy of Chinese Medical Sciences, Suzhou, China; 3 Department of Breast Surgery, Shanxi Province Cancer Hospital/Shanxi Hospital Affiliated to Cancer Hospital, Chinese Academy of Medical Sciences/Cancer Hospital Affiliated to Shanxi Medical University, Taiyuan, China; 4 Graduate School, Shanghai University of Traditional Chinese Medicine, Shanghai, China

**Keywords:** Triple-negative breast cancer (TNBC), lactylation, immune infiltration, prognosis, differentially expressed genes

## Abstract

**Background** Triple-negative breast cancer (TNBC) is an aggressive subtype of breast malignancy characterized by poor clinical outcomes and limited therapeutic options. The identification of reliable biomarkers for predicting prognosis and immunotherapeutic response remains an urgent clinical need. This study aimed to develop an integrative lactylation-related gene signature to simultaneously evaluate prognostic trajectories and immunotherapeutic sensitivity in TNBC. **Methods** Transcriptomic and clinical data from public TNBC cohorts were systematically analyzed. Lactylation-related gene signatures were used to stratify patients via consensus clustering. A scoring model was constructed based on differentially expressed genes between clusters, and its associations with immune infiltration, pathway enrichment, drug sensitivity, and clinical outcomes were evaluated. Finally, quantitative real-time polymerase chain reaction, Western blot and Confocal immunofluorescence Microscopy were used to validate the hub genes. **Results** Significant gene expression differences stratified TNBC patients into high- and low-score groups, with the high-score group demonstrating superior clinical outcomes. These patients also showed better responses to immunotherapy, as indicated by immune checkpoint profiles and chemotherapy sensitivity. Experimental validation confirmed Programmed Cell Death 1 Ligand 2, Immunoglobulin J Chain, and Colony Stimulating Factor 2 Receptor Beta as key molecular nodes. Our scoring model predicts immunotherapy efficacy, and these three genes may represent potential candidates for further therapeutic exploration in TNBC. **Conclusions** This study establishes a novel lactylation-related gene signature that effectively predicts both prognosis and immunotherapeutic sensitivity in TNBC. The identified hub genes represent promising biomarkers and potential therapeutic targets warranting further investigation.

## 1 Introduction

Globally, breast cancer (BC) represents a leading malignancy, with incidence rates demonstrating a persistent upward trajectory. Global cancer registry data from 2020 recorded 2.3 million incident cases of breast carcinoma, translating to a lifetime risk probability of 8.3% for women by age 75 (1 in 12 individuals). Projection models indicate this burden will escalate to 3 million annual diagnoses by 2040, reflecting a concerning 30.4% increase in incidence rates [1]. Triple negative breast cancer (TNBC) is characterized as a biologically unique BC classification. This malignancy is pathologically characterized by aggressive local invasion, early systemic dissemination, and unfavorable clinical outcomes. Wang et al. [2] found that in the past, tumor dimension, the number of lymphatic dissemination and the degree of histological differentiation were important factors in the treatment and prognosis of TNBC. However, clinical practice has found that due to its unique molecular profile characterized by hormone receptor negativity and targetable pathway deficiencies, TNBC exhibits intrinsic resistance to both endocrine and molecularly targeted interventions. Current postoperative chemotherapy approaches exhibit limited clinical efficacy for adjuvant disease control [3,4], which often leads to tumor recurrence. Importantly, the standard TNBC therapy is still lacking. Recent studies have explored innovative therapeutic strategies for TNBC, including metabolic reprogramming and nanomedicine-based immunomodulatory approaches [5,6].

As the end-product of anaerobic glycolysis, lactate is predominantly generated through cytosolic glucose catabolism. Ippolito et al. [7] has reshaped our understanding of lactate, revealing its multifaceted roles in energetic metabolism, immunomodulation, and cell-cell signaling beyond its classical metabolic byproduct identity. Upon oncogenic activation of aerobic glycolysis, they continuously take up large amounts of glucose and produce substantial amounts of lactate. Neoplastic cells export lactate via membrane transport systems, creating a proton-rich extracellular milieu characteristic of tumor ecosystems [8]. Additionally, molecular studies have unequivocally demonstrated lactate’s critical participation in modifying histone lysine residues. Histone lactylation is widely present in various normal and cancerous human tissues and has been found to be significant in promoting metabolic reprogramming, altering the immune environment, controlling cell fate, and influencing cancer stem cells and aging [9]. In this study, we focused on lactylation-related genes to investigate their prognostic and immunotherapeutic implications in TNBC. Clustering genes based on lactylation modification is effective because lactylation, as a polypeptide chain alteration, can influence protein function, transcriptional activity, and cellular function, consequently regulating specific biological processes or functions. Furthermore, lactylation modifications are associated with cellular metabolic states and cancer. By analyzing the expression patterns of lactylation-related genes, researchers can uncover their roles in gene regulation and cancer progression [10]. While lactylation has emerged as a significant post-translational modification, its investigation in TNBC remains notably underexplored, with scarce literature addressing this specific molecular mechanism.

Despite the emerging roles of lactylation in metabolic reprogramming and immune regulation, its involvement in TNBC remains largely unexplored. Specifically, it is unknown whether lactylation-related genes can stratify TNBC patients into distinct prognostic subgroups or reflect the tumor immune microenvironment. Given that lactate accumulation in the tumor microenvironment is known to influence immune cell function, we hypothesized that lactylation-associated gene signatures might serve as potential biomarkers for predicting immunotherapy response in TNBC. However, systematic analyses addressing this hypothesis are currently lacking.

In this study, we aimed to investigate whether lactylation-related gene signatures could stratify TNBC patients into distinct prognostic subgroups and serve as predictive biomarkers for immunotherapeutic response. We hypothesized that a lactylation-associated gene model could simultaneously evaluate prognostic trajectories and identify patients likely to benefit from immunotherapy.

## 2 Materials and Methods

This investigation sought to discover novel immunotherapeutic targets specific to TNBC. To achieve this, we employed the protocol established by Zhang et al. [11], Wang et al. [12], and then made some adjustments according to the characteristics of our research. The detailed methods are described as follows.

### 2.1 Data Retrieval and Processing

Gene expression data of TNBC patients were obtained from the TCGA database (https://xenabrowser.net/datapages/) and the GEO database (https://www.ncbi.nlm.nih.gov/geo/, accession: GSE58812). A total of 247 TNBC tissue samples and 16,915 genes were included. From the TCGA database, we also obtained relevant clinical data of the TNBC patients. To remove batch effects between the TCGA and GEO datasets, we applied the ComBat function from the ‘sva’ R package (version 3.40.0). The batch-adjusted expression data were then normalized using the ‘limma’ R package (version 3.48.3). All default parameters were used for ComBat, with the batch variable set to the data source (TCGA vs. GEO). Lactylation-related genes were curated from the literature [12,13,14,15] that reported lactylation modification in cancer. After merging and removing duplicates, a total of 336 lactylation-related genes were obtained for further analysis.

### 2.2 Screening of Prognostic Lactylation-Related Genes in TNBC

To identify prognostic lactylation-associated genes (LAGs), we performed Spearman’s rank correlation and univariate Cox analyses on 336 candidates (299 available in our data). This screening identified 18 significant genes with *p* < 0.05 in univariate Cox regression analysis, for which we conducted Kaplan-Meier survival analysis. Patients were stratified into high- and low-expression groups based on the median gene expression level, with overall survival (OS) as the endpoint. Survival differences between groups were assessed using the log-rank test, and curves were generated using the survminer R package (version 0.4.9).

### 2.3 Cluster Analysis

Based on univariate Cox regression analysis, 18 prognostic lactylation-related genes (*p* < 0.05) were identified. Given that lactylation modifications are known to regulate immune cell function and inflammatory responses within the tumor microenvironment, these prognostic genes were hypothesized to reflect distinct immune activation states. Inflammation-associated molecular subtypes were then delineated using consensus clustering with the ConsensusClusterPlus R package (version 1.62.0). The optimal number of clusters (k = 2) was determined based on the cumulative distribution function (CDF) curve and the proportion of ambiguous clustering (PAC) algorithm. Clustering was performed using 1000 resampling iterations (reps = 1000) with 80% sample resampling (pItem = 0.8), hierarchical clustering algorithm (clusterAlg = “hc”), and Pearson correlation as the distance metric (distance = “pearson”). Kaplan–Meier survival analysis was employed to evaluate survival differences between subtypes, with OS as the endpoint. Patients were stratified according to their assigned molecular subtypes, and survival curves were compared using the log-rank test. The associations between molecular subtypes and clinical characteristics (including age, clinical stage, T stage, N stage, and M stage) were visualized in a heatmap of clinical annotations alongside gene expression profiles and assessed using chi-square tests. to compare categorical variable distributions between subtypes.

### 2.4 Gene Functional Enrichment Analysis

Identification of prognostic lactylation-related genes was performed using univariate Cox regression analysis, with each gene evaluated individually as a continuous variable. Genes with *p* < 0.05 were considered statistically significant. Clustering of inflammation-associated molecular subtypes was conducted with the ConsensusClusterPlus package (version 1.62.0). Assessment of survival differences between subtypes was carried out via Kaplan-Meier analysis, and correlations with clinical characteristics were visualized in heatmaps and evaluated using chi-square tests, with *p* values adjusted for multiple comparisons using the Benjamini-Hochberg false discovery rate (FDR) method.

### 2.5 Construction of Lactic Acid Scoring Model, Compare the Difference of Immune Cell Infiltration

Gene expression dimensionality reduction was conducted via principal component analysis (PCA), using the first two components (PC1 and PC2) as representative features for signature scoring. Following this, the ESTIMATE algorithm was applied to quantify the tumor microenvironment, from which composite, stromal, and immune scores were derived [16]. A panel of 29 Functional gene expression signatures (Fges) derived from Bagaev et al. (Cancer Cell, 2021) were employed to comprehensively characterize the immune landscape [17]. The infiltration levels of 23 immune cell types were quantitatively assessed using single-sample GSEA (ssGSEA), which was performed with the R package “GSVA” (version 1.40.1). The gene set markers for these 23 immune cell types were derived from the built-in immune cell signatures provided in the GSVA package [18].

### 2.6 Differential Expressed Genes Analysis

Differential expression analysis between the C1 and C2 clusters was performed using the limma R package. Genes with (|Log_2_Fold Change (FC)| > 1 and FDR < 0.05) were considered significantly differentially expressed. *p*-values were adjusted for multiple testing using the Benjamini-Hochberg method. Functional enrichment analysis of these genes was conducted using the “clusterProfiler” R package (v4.0.4) for GO and KEGG terms, The background gene set consisted of all protein-coding genes in the human genome (org.Hs.eg.db). Enriched GO terms and KEGG pathways were identified using a hypergeometric test, and *p*-values were adjusted for multiple testing using the Benjamini-Hochberg method. Terms with an adjusted *p*-value < 0.05 were considered statistically significant. The top 20 genes ranked by *p*-value from univariate Cox regression were selected for further investigation, as this number provided a balance between model parsimony and predictive performance while ensuring all selected genes met the significance threshold (*p* < 0.05). The top 20 genes were subjected to unsupervised hierarchical clustering to stratify patients into two geneClusters. Survival differences between resulting gene clusters were evaluated by Kaplan-Meier analysis with the log-rank test. To further assess the independent prognostic value of the gene signature, multivariable Cox regression analysis was performed, adjusting for clinical confounders including age, clinical stage, T stage, N stage, and M stage. Associations with clinical parameters were visualized in a heatmap and statistically validated by chi-square tests.

### 2.7 Prediction of Curative Effect of Immunotherapy

To evaluate the immunosuppressive landscape, we compared the expression levels of five key immunoinhibitors (CD274, CTLA4, LAG3, PDCD1, TIGIT) across the predefined molecular subgroups and risk categories. The Immunophenotype Score (IPS) from the TCIA database was utilized to predict immunotherapy response in TCGA-BRCA patients The IPS integrates four major categories of immune-related features—immune cell infiltration composition, MHC molecule expression, immunostimulatory molecules, and immunosuppressive molecules—into a 0–10 scoring scheme using a machine learning approach, with higher scores indicating greater tumor immunogenicity and predicted response to immune checkpoint blockade [18]. These predictions were subsequently validated in two independent immunotherapy cohorts: GSE135222 (advanced NSCLC treated with PD-1/PD-L1 inhibitors) and GSE91061 (melanoma treated with Nivolumab).

### 2.8 Prediction of Drug Sensitivity in Non-Immunotherapy

We used “pRRophetic” package of R (version 0.5.0; R version 4.0.1). This package implements ridge regression models trained on gene expression and drug sensitivity data from the Cancer Genome Project (CGP) database, encompassing over 700 cancer cell lines. The half-maximal inhibitory concentration (IC50) was estimated for each sample as the drug response metric, with lower IC50 values indicating greater sensitivity. Differences in estimated IC50 values between the high- and low-score groups were compared using the Wilcoxon rank-sum test (*p* < 0.05).

### 2.9 Gene Mutation Analysis

Utilizing TCGA somatic mutation datasets, we performed mutational analysis via the maftools R package (version 2.14.0). Specifically, mutation profiles were visualized using oncoplots to display the landscape of the top 20 most frequently mutated genes. Tumor mutational burden (TMB) was calculated as the total number of non-synonymous mutations per sample and compared between the high- and low-score groups using the Wilcoxon rank-sum test. Additionally, the differential mutation frequencies of individual genes between the two score groups were assessed using Fisher’s exact test, and mutually exclusive or co-occurring mutation patterns were evaluated using the somaticInteractions function.

### 2.10 Copy Number Variation Analysis

Leveraging the GSCA database (Gene Set Cancer Analysis; http://bioinfo.life.hust.edu.cn/GSCA; 2023 version), we conducted an integrated multi-omics analysis. Specifically, the “CNV & Expression” module was used to assess the correlation between heterozygous/homozygous copy number variations (CNVs) and mRNA levels. Spearman’s rank correlation coefficient was calculated, and raw *p*-values were adjusted for multiple testing using the Benjamini-Hochberg false discovery rate (FDR) method (FDR < 0.05). The “Methylation & Expression” module was employed to evaluate associations between mRNA expression and DNA methylation. Methylation levels for each gene were quantified using beta-values, which represent the ratio of methylated probe intensity to the total probe intensity, ranging from 0 (unmethylated) to 1 (fully methylated). Additionally, the “SNV Summary” module was utilized to determine the proportion of single nucleotide variations in the differentially expressed genes of interest.

### 2.11 Cell Culture

The human normal breast cell line MCF-10A (GNHu50) and TNBC cell lines, MDA-MB-231 (TCHu227) and BT-549 (TCHu 93) were sourced from the Cell Bank of the Chinese Academy of Sciences (Shanghai, China). All cell lines were authenticated by short tandem repeat (STR) profiling, and certificates were provided by the cell bank. Before use in this study, all cell lines were tested negative for mycoplasma contamination using a PCR-based mycoplasma detection kit (Beyotime, C0301S, Shanghai, China). Cells were cultured as follows: MCF-10A in its specific complete medium (Pricella, CM-0525, Wuhan, Hubei, China). According to the manufacturer’s formulation, this complete medium consists of DMEM/F12 basal medium supplemented with 5% horse serum, 20 ng/mL epidermal growth factor (EGF), 0.5 μg/mL hydrocortisone, 10 μg/mL insulin, and 100 ng/mL cholera toxin, BT-549 in Roswell Park Memorial Institute (RPMI) 1640 medium (Gibco, C11875500BT, Grand Island, NY, USA), and MDA-MB-231 in Dulbecco’s Modified Eagle’s Medium (DMEM) (Gibco, C11995500BT, Grand Island, NY, USA). All Gibco-based media were uniformly supplemented with 10% fetal bovine serum (HyClone, SV30208.02, Logan, UT, USA) and 1% penicillin/streptomycin (Solarbio, P1400, Beijing, China). All cells were incubated at 37°C in a 5% CO_2_ environment.

### 2.12 RNA Extraction and qRT-PCR

Total RNA was extracted from both normal and TNBC cell lines using TRIzol Reagent (Invitrogen, 15596026, Carlsbad, CA, USA). RNA concentration and purity were assessed using a NanoDrop 2000 spectrophotometer (Thermo Fisher Scientific, Waltham, MA, USA), with A260/A280 ratios between 1.8 and 2.0 considered acceptable for further analysis. cDNA was synthesized from 1 μg of total RNA using the Evo M-MLV RT Mix Kit (AGbio, AG11728, Changsha, Hunan, China) with gDNA Clean for qPCR Ver.2 according to the manufacturer’s instructions. Quantitative real-time PCR was performed using SYBR Green Premix Pro Taq HS qPCR Kit (AGbio, AG11701, Changsha, Hunan, China) on a StepOnePlus Real-Time PCR System. Each 20 μL reaction contained 10 μL of SYBR Green Premix, 0.4 μL of each forward and reverse primer (10 μM stock, final concentration 0.2 μM), 2 μL of diluted cDNA template, and 7.2 μL of nuclease-free water. Amplification was performed over 40 cycles under the following conditions: 95°C for 15 s (denaturation), 60°C for 15 s (annealing), and 72°C for 45 s (extension). Melt curve analysis was performed after amplification to confirm reaction specificity, with a single distinct peak indicating specific amplification. The 2^−ΔΔCt^ method was applied to calculate relative mRNA expression levels normalized to β-actin, utilizing ABI StepOne Software v2.1 (Applied Biosystems, Foster City, CA, USA). All experiments included three biological replicates, and results are expressed as fold changes. The primer sequences used for qRT-PCR are listed in Table 1.

**Table 1:** Primers used for qRT-PCR.

Gene	Species	Primer Sequence (5′ to 3′)
*XCL1*	human	Forward: TGGCTAGTGTCTATCAGAGGTG
		Reverse: TGGACCCTCAGAATGGGAAC
*PDCD1LG2*	human	Forward: ACCAGTGTTCTGCGCCTAAA
		Reverse: GCACTGTTCACTTCCCTCTTTG
*GZMB*	human	Forward: CCCTGGGAAAACACTCACACA
		Reverse: GCACAACTCAATGGTACTGTCG
*IFNG*	human	Forward: TCGGTAACTGACTTGAATGTCCA
		Reverse: TCGCTTCCCTGTTTTAGCTGC
*IL18RAP*	human	Forward: TTGAGCTGGTGTAACGTGGCT
		Reverse: GGCTACACCTTCAGCTGTCTCTTT
*GNLY*	human	Forward: GGCTCCCTGCCCATAAAACA
		Reverse: CTTGTTCTCTCACCAGCCCC
*TRIM22*	human	Forward: TATCCAGATCGAGAGACAGAAGA
		Reverse: TGTCCAGCTTTCACTCCTTTT
*IKZF3*	human	Forward: TCGGAGATGGTTCCAGTTATCA
		Reverse: ATTCTGGCGTTCTTCATGGTT
*IGJ (JCHAIN)*	human	Forward: CAGCCTTAACCCCAGATGCC
		Reverse: GGTGGCAGGGAGTTGGTTTT
*UBASH3A*	human	Forward: AACAAGCTCAAGAGCCGCA
		Reverse: AGTGTCACGTGTGGGAAGAC
*S1PR4*	human	Forward: AAGTTGCAGTCTTGCGTGTG
		Reverse: GTTCCCTGCTCCCCATACAG
*GPR171*	human	Forward: CTGCTGCGGCTAACCAAAC
		Reverse: CTGGGCAGAAGAACGAACTG
*CD274*	human	Forward: GCAGGGCATTCCAGAAAGATG
		Reverse: TAGGTCCTTGGGAACCGTGA
*TRAT1*	human	Forward: ATTGTTGGGCTTGGCTTTGG
		Reverse: ATCAAGTGAGGCGTAGCACA
*CD96*	human	Forward: CACGGATTCTTGGGTCCTTCT
		Reverse: AACTTCCGCCCATCATCGAA
*CXCL13*	human	Forward: TCAGCAGCCTCTCTCCAGT
		Reverse: CTTGGACAACCATTCCCACG
*CXCR6*	human	Forward: AGCAAGCTCATCTCTGGAACAA
		Reverse: CATGCTCTGCCATGGTGTCT
*CSF2RB*	human	Forward: ATTCTACAAGCCCAGCCCAG
		Reverse: CCTTGGCTGAACAGAGACGA
*EMB*	human	Forward: TTTCTTCAGCGTCCTACCCG
		Reverse: TAAAAGGCGAATCTGGGGCA
*STAP1*	human	Forward: CAAAACGTGTCACTCCTACCTG
		Reverse: CGGACGTACTCTGCTCTGTC
*β-actin*	human	Forward: CACAGAGCCTCGCCTTTGC
		Reverse: AATCCTTCTGACCCATGCCC

### 2.13 Western Blot Analysis

Protein concentration was determined using a bicinchoninic acid (BCA) protein assay kit (Beyotime, P0012). Cell and tissue lysates (20 μg) were resolved on 8% SDS-PAGE gels and transferred to 0.2 μm PVDF membranes. The membranes were blocked with 5% non-fat milk (90 min, room temperature [RT]) and subsequently incubated at 4°C overnight with specific primary antibodies: PDCD1LG2 (Proteintech, 27406-1-AP, Rosemont, IL, USA; 1:600), IGJ (Abcam, ab269855, Cambridge, MA, USA; 1:600), CSF2RB (Affinity Biosciences, DF7680, Liyang, Jiangsu, China; 1:1000), and GAPDH (Abcam, ab181602, Cambridge, MA, USA; 1:1000). Following primary antibody incubation, membranes were washed and exposed to HRP-conjugated goat anti-rabbit IgG (H + L) secondary antibody (Beyotime, A0208; 1:5000) for 2 h at room temperature. Signal detection was performed with BeyoECL Star chemiluminescent substrate (Beyotime, P0018AS), and band intensities were quantified using Image Lab software (version 5.0, Bio-Rad, Hercules, CA, USA).

### 2.14 Confocal Immunofluorescence

Macrophages of different phenotypes were plated on 35 mm glass-bottom confocal dishes (MatTek, Ashland, MA, USA) at a density of 3 × 10^5^ cells per dish, fixed with 4% paraformaldehyde, and permeabilized with 0.3% Triton X-100 (Beyotime, ST797). After blocking with 5% BSA (Beyotime, ST025) for 30 min at RT, the cells were incubated with primary antibodies against PDCD1LG2 (Proteintech, 27406-1-AP; 1:200), IGJ (Abcam, ab269855; 1:200), and CSF2RB (Affinity Biosciences, DF7680; 1:200), followed by corresponding FITC-conjugated goat anti-rabbit IgG (H + L) secondary antibody (Invitrogen, 65-6111, Carlsbad, CA, USA; 1:50) or Alexa Fluor 594-conjugated goat anti-rabbit IgG (H + L) cross-adsorbed secondary antibody (Invitrogen, A-11012, 1:50) and DAPI (Beyotime, C1005; 1:5000). Images were acquired using a Zeiss LSM-800 confocal microscope (Carl Zeiss AG, Oberkochen, Germany) and quantified with Fiji (ImageJ, version 2.3.0/1.53q, National Institutes of Health, Bethesda, MD, USA).

### 2.15 Statistical Analysis

Statistical analyses were conducted in R 4.0.1 and GraphPad Prism 8 (GraphPad Software, San Diego, CA, USA). We employed the Wilcoxon rank-sum test for gene expression comparisons. To account for multiple testing, raw *p*-values were adjusted using the Benjamini-Hochberg FDR method, with adjusted *p* < 0.05 considered statistically significant. Log-rank tests with KM curves for survival analysis, and Spearman’s method for correlation assessments. Independent prognostic factors were identified via multivariable Cox regression, which computed hazard ratios (HR) and corresponding 95% confidence intervals (CI). The following covariates were included in the multivariable model: age, clinical stage, T stage, N stage, and M stage. Patients with missing data for any of these covariates were excluded from the multivariable analysis. The proportional hazards assumption was tested using Schoenfeld residuals, and no significant violations were detected (all *p* > 0.05). A statistical difference was considered to be significant as *p < 0.05, **p < 0.01 and ***p < 0.001. ns means *p* > 0.05. Due to the limited sample size of TNBC cases, we did not split the data into separate training and validation cohorts. Instead, we used the combined dataset for all analyses, and the robustness of our findings was supported by cross-cancer validation using independent immunotherapy cohorts (GSE135222 and GSE91061).

## 3 Results

**Figure 1:**
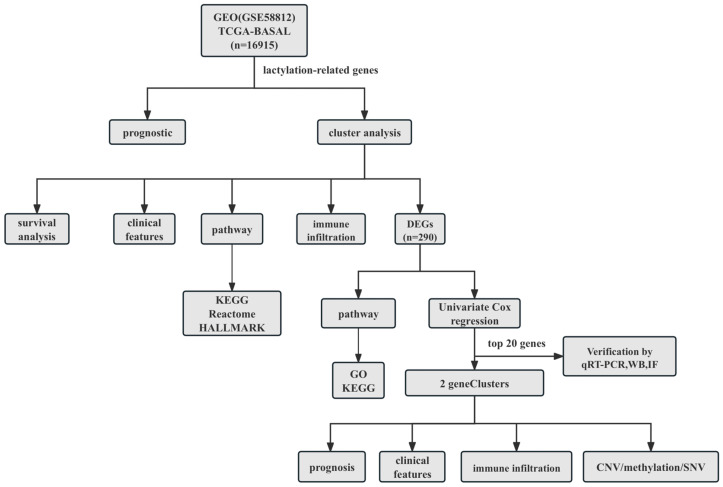
Schematic flowchart of the study design.

The flow chart of our study is shown in Fig. 1.

### 3.1 Expression of Lactylation-Related Genes in TNBC

**Figure 2:**
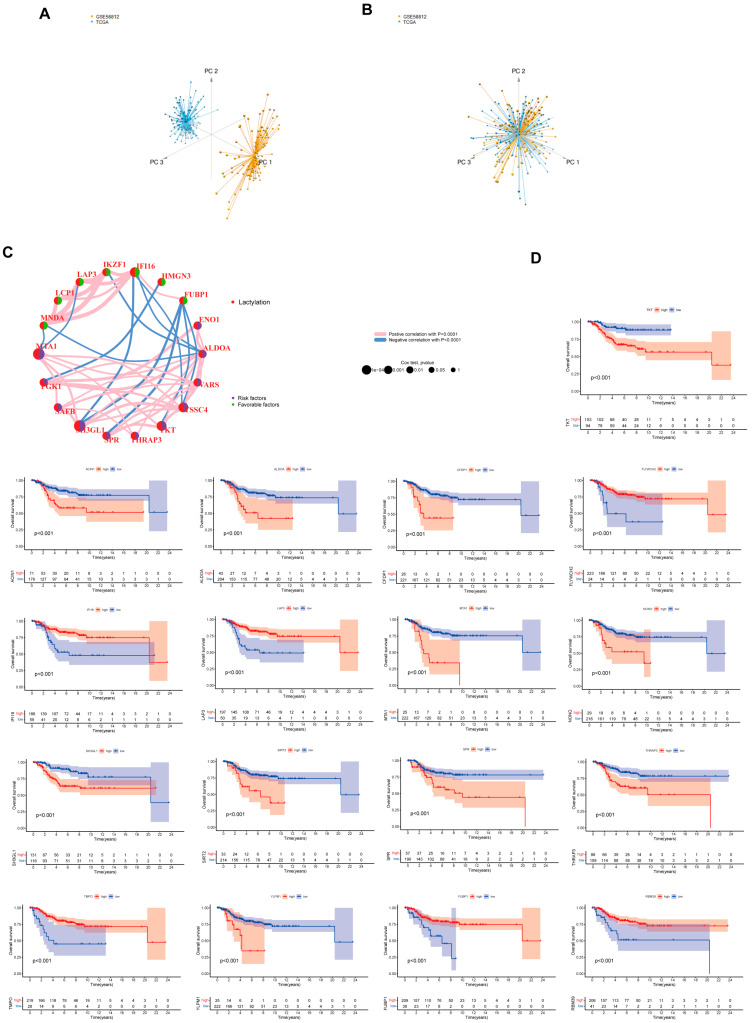
Lactylation-related gene expression in Triple-negative breast cancer (TNBC). (**A**) PCA plot before batch effect correction, showing distinct separation between GSE58812 and TCGA-BASAL samples. (**B**) PCA plot after batch effect correction, demonstrating successful integration of the two datasets. (**C**) Correlation network and univariate Cox regression of lactylation-related genes (18 genes with Cox *p* < 0.05). (**D**) Prognostic analysis of lactylation-related genes (*p* < 0.001).

Based on the GEO (GSE58812) and TCGA (TCGA-BASAL) database, 16915 genes and 247 samples were obtained. The graph on the right is the one after the batch effect that has been removed from the graph on the left (Fig. 2A,B). To characterize the correlation of lactate-induced epigenetic regulators expression, we performed correlation analysis and univariate regression analysis of lactylation-related genes (Additional file 1). There were 18 genes showed correlations (*p* < 0.0001). Among that MNDA, LCP1, LAP3, IKZF1, IFI16, HMGN3 and FUBP1 genes were identified as favorable factors, while the remaining genes were considered risk factors including ENO1, ALDOA, VARS, TSSC4, TKT, THRAP3, SPR, SH3GL1, SAFB, PGK1 and MTA1 (Fig. 2C). They regulated each other within a network, which could affected the progression of TNBC.

To clarify the interaction among lactylation-based biomarkers for TNBC survival prediction, we performed survival analysis of top 200 expressed genes in lactylation-related genes. There were 70 (*p* < 0.05) genes whose survival time was longer with higher expression. And among that the higher the expression of FLYWCH2, IFI16, LAP3, TMPO, FUBP1 and RBM39 genes (*p* < 0.001), the longer their survival (Fig. 2D). There were 68 (*p* < 0.05) genes whose survival time was longer with lower expression (Additional file 2).

### 3.2 Evaluation of Clinical Values of Lactylation-Related Genes Subgroups in TNBC Patients Using Consensus Clustering

**Figure 3:**
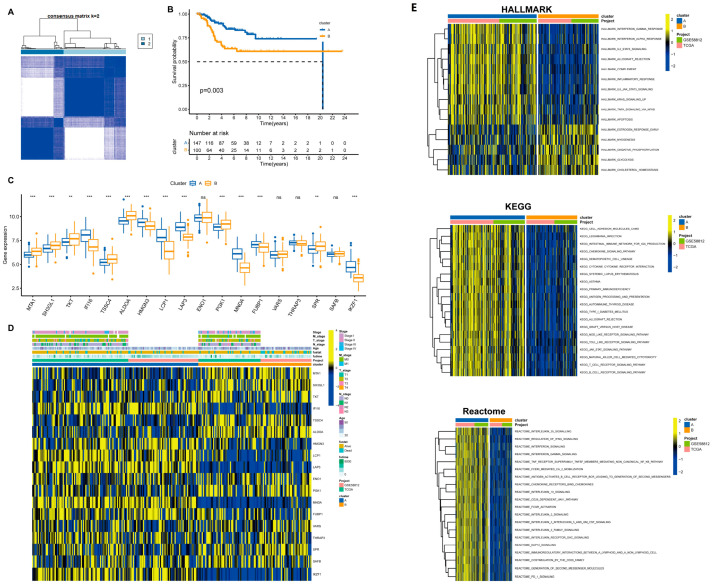
Consensus clustering of TNBC based on lactylation-related genes. (**A**) Consensus clustering matrix (k = 2). (**B**) Kaplan-Meier survival curves for the two clusters. (**C**) Expression levels of lactylation-related genes between clusters. (**D**) Heatmap of clinical features and gene expression. (**E**) GSEA enrichment results for KEGG, Reactome, and HALLMARK pathways. ns no significance, **p < 0.01, ***p < 0.001.

Lactylation-based gene expression profiling partitioned TNBC samples into two clusters via unsupervised clustering (Fig. 3A). The two TNBC clusters exhibited significantly different survival outcomes (*p* = 0.003) (Fig. 3B). Survival outcomes were significantly inferior in Cluster B relative to Cluster A. Gene expression ratios of 18 candidate markers were quantified between TNBC clusters through ssGSEA. The two TNBC clusters demonstrated distinct immunogenic signatures, characterized by differential enrichment of 18 functionally diverse immune cell types (Fig. 3C). In addition, there was a prognostically impactful dichotomy between TNBC patients in the two cluster groups in terms of clinical stage, M stage, T stage, N stage, age, fustat, and futime status (Fig. 3D).

Subsequently, for purpose of compare the differences in signal pathways between cluster A and cluster B, we downloaded the HALLMARK, KEGG and Reactom signal pathway from the Molecular Signatures Database (MSigDB www.Gsea-msigdb.Org/gsea/msigdb). Then GSEA was performed (Fig. 3E). Cluster A demonstrated predominant enrichment of interferon-related signaling and STAT-mediated transcriptional regulation in HALLMARK pathway analysis such as INTERFERON_GAMMA_RESPONSE, INTERFERON_ALPHA_RESPONSE and IL2_STAT5_SIGNALING, cluster B was enriched in early estrogen reaction, myogenesis and glycolysis, such as ESTROGEN_RESPONSE_EARLY, MYOGENESIS and GLYCOLYSIS. Besides, Cluster A exhibited predominant enrichment of KEGG pathways related to cell adhesion molecules, chemokine and cytokines, such as CELL_ADHESION_MOLECULES_CAMS, CHEMOKINE_SIGNALING_PATHWAY and CYTOKINE_CYTOKINE_RECEPTOR_INTERACTION whlie Reactome-enriched pathways in cluster A was mostly focused on interleukin, interferon and chemokine, such as INTERLEUKIN_35_SIGNALLING, INTERFERON_SIGNALING and CHEMOKINE_RECEPTORS_BIND_CHEMOKINES.

### 3.3 Constructed Lactylation Score Model and Explored Immune Infiltration among the Two Distinct Clusters

**Figure 4:**
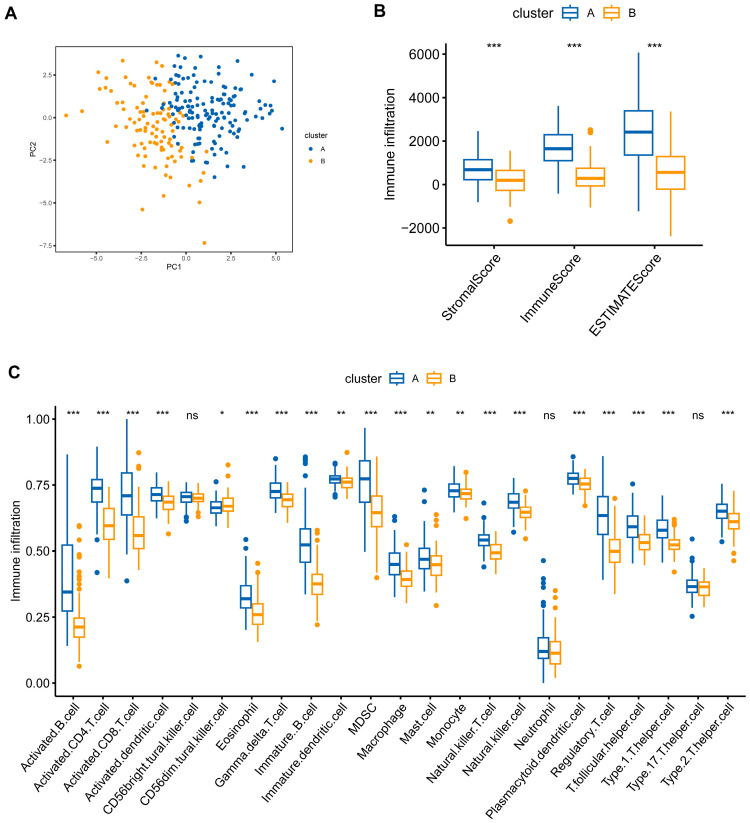
Correlation between cluster groups and immune cell infiltration in TNBC. (**A**) Principal component analysis (PCA). (**B**) Comparison of ESTIMATE algorithm scores (StromalScore, ImmuneScore, ESTIMATEScore) between cluster A and cluster B. Higher scores indicate greater infiltration of stromal and immune cells, corresponding to lower tumor purity. (**C**) Differences in immune cell infiltration scores between the two clusters assessed by ssGSEA. ns no significance, *p < 0.05, **p < 0.01, ***p < 0.001.

PCA of TNBC samples demonstrated that it could be divided into two clusters (Fig. 4A). Fig. 4B demonstrated that cluster A had a higher StromalScore, ImmuneScore, and ESTIMATEScore, indicating greater infiltration of immune and stromal cells, and thus lower tumor purity in cluster A compared to cluster B. Subsequently, we calculated the immune infiltration between the two clusters, and the results interpreted that the Cluster A had a taller immune infiltration score than Cluster B, except CD56dim. natural killer. cell (Fig. 4C).

### 3.4 Identification of Differential Genes and Biological Function Analysis between the Cluster A and Cluster B

**Figure 5:**
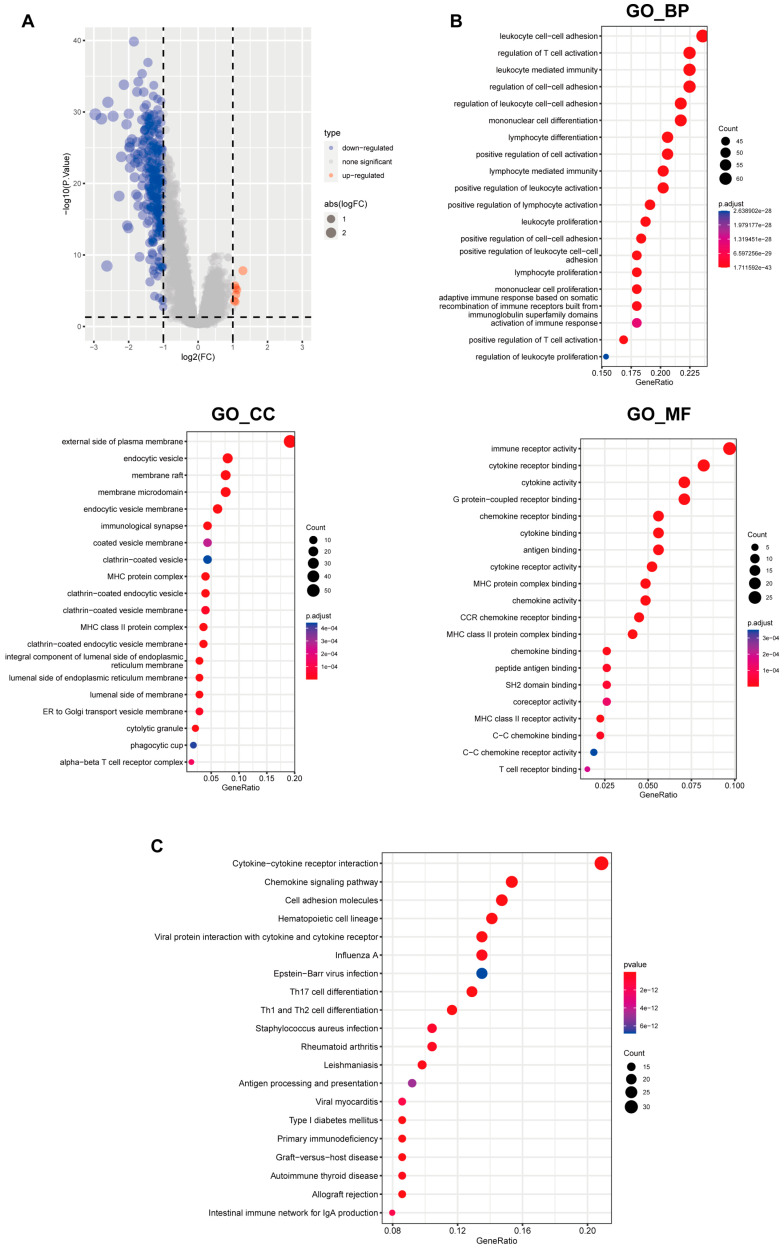
Identification of DEGs between clusters. (**A**) Volcano plot of DEGs. (**B**) GO enrichment analysis (BP: Biological Process, CC: Cellular Component, MF: Molecular Function). (**C**) KEGG pathway enrichment analysis.

Taking |log_2_ FC| > 1 and adjusted *p* < 0.05 as the stringent selecting criterion, there were a total of 290 DEGs in Cluster A versus Cluster B (Fig. 5A, Additional file 3). GO and KEGG pathway analyses of the 290 DEGs identified significant enrichment in immune-related processes, including leukocyte adhesion, T cell activation regulation, and leukocyte-mediated immunity (Fig. 5B). Moreover, KEGG indicated that DEGs were also functionally concentrated in cytokine-cytokine receptor interaction, chemokine signaling pathway and cell adhesion molecules (Fig. 5C).

### 3.5 Estimated the Clinical Values of DEGs in TNBC Patients between Two Distinct Gene Clusters

**Figure 6:**
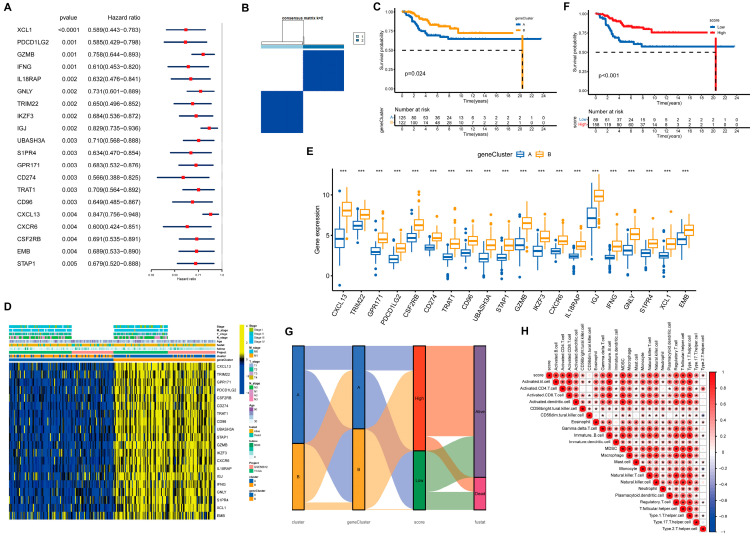
Prognostic gene signature construction. (**A**) Top 20 prognostic DEGs by univariate Cox regression. (**B**) Consensus clustering of 20 genes (k = 2). (**C**) Survival curves for two gene Clusters. (**D**) Heatmap of clinical features. (**E**) Expression of 20 genes between gene Clusters. (**F**) Survival curves for high- and low-score groups. (**G**) Sankey diagram of cluster, geneCluster, score, and survival status. (**H**) Correlation between score and immune cell infiltration. The score for each patient was calculated as the sum of PC1 and PC2 derived from principal component analysis of the 20 prognostic DEGs. Patients were stratified into high- and low-score groups using the median score as the cutoff. *p < 0.05; ***p < 0.001.

The 290 DEGs were subjected to univariate Cox regression analysis to evaluate their survival predictive value. The top 20 most significant genes underwent rigorous selection for subsequent investigation according to *p* < 0.05 (Fig. 6A). We found that it was appropriate to divide the 20 genes into two geneClusters (Fig. 6B). Survival analysis showed better prognosis for geneCluster B than geneCluster A (Fig. 6C). Simultaneously, it was conspicuous that there was a statistically significant divergence among TNBC patients in the two geneCluster groups in respect to clinical stage, M stage, T stage, N stage, age, fustat, and futime status (Fig. 6D). Afterwards, We calculated the differential gene expression between the two geneClusters and the results showed that the gene expression level (*p* < 0.001) of geneCluster A was significantly lower than geneCluster B (Fig. 6E). We used PCA to calculate the score (PC1 + PC2) based on 20 genes, and analyzed prognostic stratification by risk scoring. And we demonstrated that the higher the score, the better the prognosis (Fig. 6F). Furthermore, the Sankey diagram was constructed to show the relationship between clusters, score, and prognosis. The results showed that geneCluster B mainly comes from cluster A, which had the characteristics of a high score and the fustat is alive (Fig. 6G). Moreover, we found a significant association was observed between most of the immune infiltrating cells according to Fig. 6H (Additional file 4).

**Figure 7:**
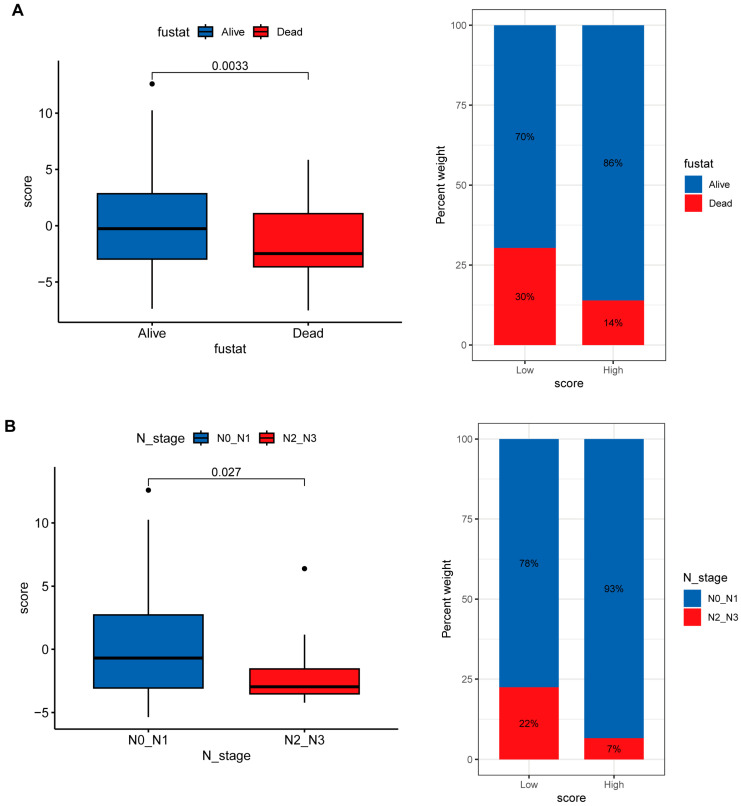
Clinical associations of the scoring model. (**A**) Survival outcomes in high- vs. low-score groups. (**B**) N stage distribution between score groups. High- and low-score groups were defined based on the median score as described in Fig. 6.

**Figure 8:**
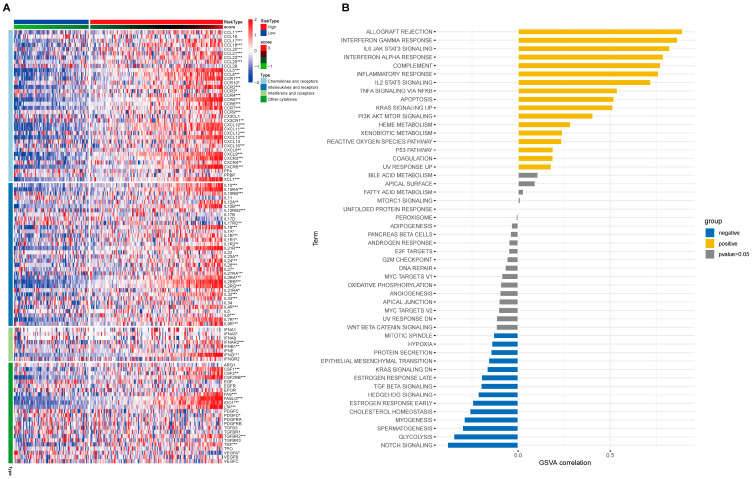
Molecular characteristics of score groups. (**A**) Differential expression of chemokines, receptors, and pro-oncogenic factors. (**B**) Correlation between score and 50 HALLMARK pathways by GSVA. Score calculation and group cutoffs are detailed in Fig. 6.

Based on the above findings, it prompted us to systematically investigate the association between geneCluster and survival state. The analysis revealed that the group with high score had a good survival state and low N stage (Fig. 7A,B). Of note, significant differences in chemokine, interleukin and interferon were also exhibited between the high- and low-score groups (Additional file 5). Our results demonstrated that a higher score correlated with increased expression of certain chemokines, such as CCL11, CCL17, and CCL18 (Fig. 8A). To demonstrated the association of pathways with genes, we did a HALLMARK score, performed a GSVA correlation analysis of 50 pathways, and found a positive correlation with ALLOGRAFT REJECTION, INTERFERON GAMMA RESPONSE, IL6 JAK STST3 SIGNALING, etc. and negetive correlation with NOTCH SIGNALING, GLYCOLYSIS, SPERMATOGENESIS, etc., while no correlation with BILE ACID METABOLISM, APICAL SURFACE, FATTY ACID METABOLISM, etc. (Fig. 8B).

### 3.6 Evaluated the Efficacy of Immunotherapy and Non-Immunotherapy Drugs of This Score Model

**Figure 9:**
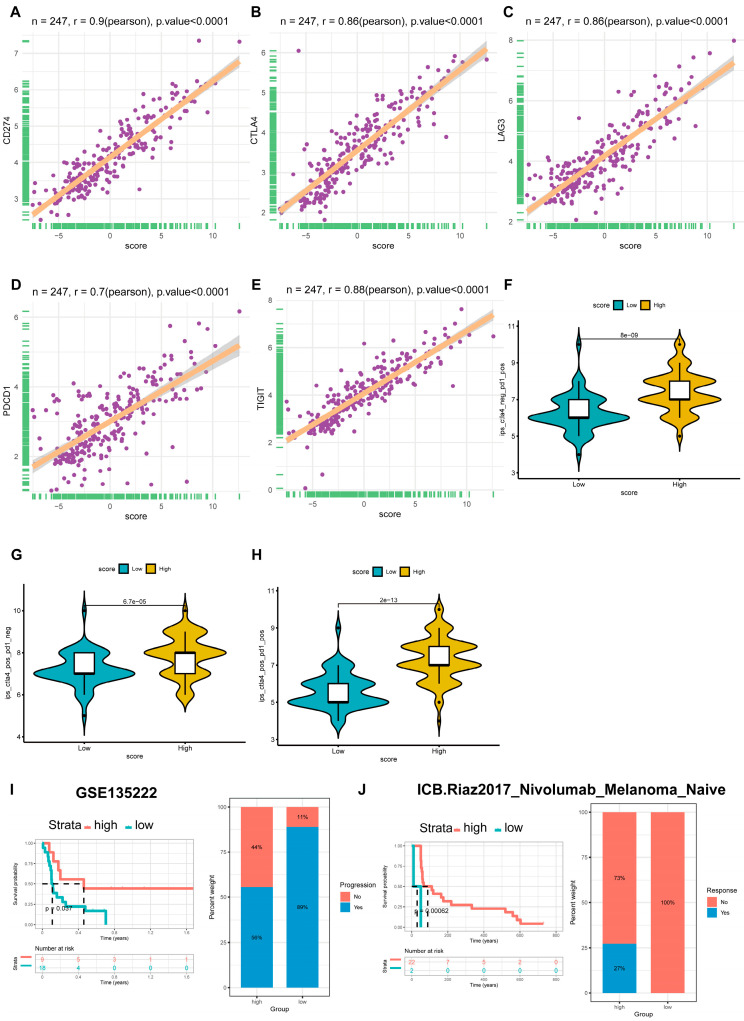
Immunotherapy response prediction. (**A**) Correlation between risk score and CD274 expression. (**B**) Correlation between risk score and CTLA4 expression. (**C**) Correlation between risk score and LAG3 expression. (**D**) Correlation between risk score and PDCD1 expression. (**E**) Correlation between risk score and TIGIT expression. (**F**) IPS score for CTLA4-negative/PD-1-positive blockade. (**G**) IPS score for CTLA4-positive/PD-1-negative blockade. (**H**) IPS score for CTLA4-positive/PD-1-positive blockade. (**I**) Kaplan-Meier survival curves for high- and low-score groups in the GSE135222 cohort. (**J**) Kaplan-Meier survival curves for high- and low-score groups in the GSE91061 cohort. High- and low-score groups were defined as described in Fig. 6.

Immune checkpoint-associated genes were mechanistically implicated in modulating therapeutic responses to immunotherapy. The immunoregulatory axis comprising PD-L1, CTLA4, LAG3, PD-1 and TIGIT was transcriptionally characterized to authenticate the scoring-immunity relationship. Consequently, the higher the score, the more immune cells infiltration and the higher expression of CD274 (PD-L1), CTLA4, LAG3, PDCD1 and TIGIT, suggesting a better efficacy of immunotherapy (Fig. 9A–E). In addition, IPS scoring served as a predictive metric for assessing immunotherapy response across differentially scored patient cohorts. Our investigation demonstrated an association between the scoring system and immune checkpoint inhibitor efficacy. Fig. 9F–H depicts superior treatment response rates among high-scoring patients receiving ICIs (ctla4_neg_pd1_pos, ctla4_pos_pd1_neg, ctla4_pos_pd1_pos, *p* < 0.05). High scores correlate with potential responsiveness to PD-1/CTLA-4 inhibitors [18]. To validated the correlation between the score and immunotherapy, we conducted immunotherapy drug analysis and survival analysis on GSE135222 and ICB.Riaz2017_Nivolumab_Melanoma_Naïve, as well as an examination of the relationship between high- and low-score and the efficacy of immunotherapy. Our findings achieved the same conclusion as the scoring model, confirming that patients in the high-score category exhibited enhanced responsiveness to immunotherapeutic interventions (Fig. 9I,J).

**Figure 10:**
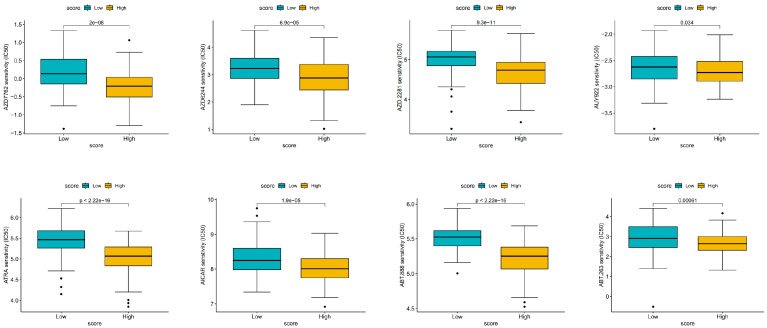
Drug sensitivity prediction for non-immunotherapy agents. Comparison of estimated IC50 values between high- and low-score groups. Drugs with lower IC50 in the high-score group (e.g., ABT.263, ABT.888, AICAR) indicate greater predicted sensitivity, whereas drugs with lower IC50 in the low-score group (e.g., A.443654, AKT inhibitor VIII) suggest reduced sensitivity in high-score patients.

Besides, we determined sample-specific IC50 values through computational analysis based on the predicted model of these 138 non-immunotherapeutic drugs by pRRophetic package (Additional file 6). There were 65 genes showed correlations in the high-score array (*p* < 0.05) while there were 33 genes demonstrated correlations in the low-score array (*p* < 0.05). A.443654, AKT.inhibitor.viii, AS601245, AZD.0530, Bexarotene, BI.2536 drugs (*p* < 0.05) were sensitive in the low score groups, while ABT.263, ABT.888, AICAR, AP.24534, ATRA and AUY922 drugs (*p* < 0.05) were sensitive in the high score groups (Fig. 10).

### 3.7 Differences in Gene Mutations between the Two Score Groups

We conducted mutation profiling to delineate genomic differences across the stratified scoring cohorts, we selected 6223 differentially mutated genes for comparison (Additional file 7). As shown in Supplementary Fig. S1, TP53 (62%), TTN (21%), FAT3 (12%), FLG (11%) and USH2A (11%) showed the greatest mutation prevalence among high-risk patients, while TP53 (62%), TTN (22%), MUC16 (18%), ANK1 (11%) and MUC17 (11%) were the top 5 genes in the low-score group. In general, the high-score group had a higher frequency of genomic mutations than low-score group (84.52% vs. 82.22%).

In order to further analyze 19 genes in TNBC, the CNV, methylation and SNV data of TNBC were obtained. Through CNV analysis, heterozygous amplifications and deletions emerged as the most common genomic aberrations in TNBC, with homozygous changes being comparatively rare (Supplementary Fig. S2A,B). Bivariate analysis demonstrated a positive association between mRNA expression and CNVs of IL18RAP, GNLY, CD274, CSF2RB, S1PR4, TRIM22 and IKZF3 in TNBC, and an inverse association for GZMB and XCL1 (Supplementary Fig. S2C). Furthermore, we found that methylation of TRAT1 demonstrated a positive association with transcriptional levels in TNBC, while the methylation of the other genes was negatively correlated with mRNA expression (Supplementary Fig. S2D). The SNV analysis revealed that UBASH3A and IKZF3 had the highest mutation frequency among TNBC samples (Supplementary Fig. S2E).

### 3.8 Verification of Diagnostic Markers

**Figure 11:**
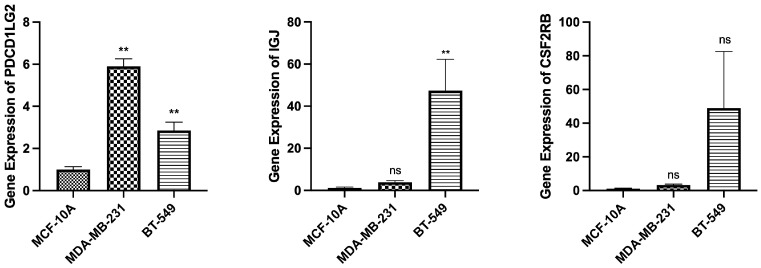
qRT-PCR validation of three hub genes (PDCD1LG2, IGJ, CSF2RB) in normal (MCF-10A) and TNBC (MDA-MB-231, BT-549) cell lines. *n* = 3. ns no significance, **p < 0.01.

**Figure 12:**
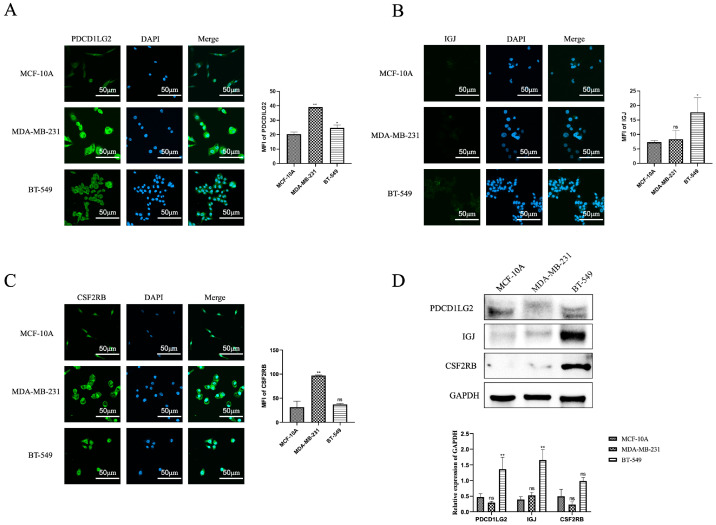
Protein expression validation of hub genes. (**A**) Confocal immunofluorescence images of PDCD1LG2. (**B**) Confocal immunofluorescence images of IGJ. (**C**) Confocal immunofluorescence images of CSF2RB. (**D**) Western blot analysis of the three hub proteins. *n* = 3. For efficient antibody probing, membranes were cut horizontally based on target protein molecular weights prior to hybridization. All membrane pieces were processed in parallel under identical conditions. ns no significance, *p < 0.05, **p < 0.01.

The 20 identified DEGs were quantitatively assessed for mRNA abundance in both normal breast and TNBC cellular populations. Among them, PDCD1LG2, IGJ and CSF2RB exhibited expression patterns consistent with the bioinformatics predictions, and their results are shown in Fig. 11. Meanwhile, we screened three genes for Western blot (WB) analysis and Confocal IF Microscopy (Fig. 12) verification. The protein expression patterns of these genes were generally consistent with the mRNA trends observed by qRT-PCR, though some quantitative differences were noted, likely reflecting post-transcriptional regulation.

## 4 Discussion

Breast cancer represents a predominant malignancy and leading oncologic mortality factor in female populations [19]. Building upon ER, PR, and HER2 expression, BC is divided into five subtypes. TNBC, accounting for 10% to 20% of invasive BC cases, is more common in young women and those with BRCA1 mutations [20,21,22]. Traditional treatment for TNBC includes surgery and adjuvant chemotherapy, but effective therapies for advanced TNBC are limited, leading to poor prognosis and unmet clinical needs [23,24]. Thus, new effective treatments for TNBC are urgently needed.

Abnormal glucose metabolism is a hallmark of tumor tissues, contributing to aggressive phenotypes. Lactate, a key component of glucose metabolism, acts as a metabolic substrate and redox signaling molecule. In TNBC, abnormalities in glucose metabolism and lactate production, along with lactylation modifications, are linked to poor prognosis, genomic instability, treatment resistance, and immunosuppression [25]. Understanding lactylation-related gene expression in TNBC may offer new therapeutic strategies.

To investigate lactylation modification in TNBC, we analyzed TCGA and GEO datasets, identifying 18 lactate-associated genes that are linked to prognosis and revealing the existence of two distinct clusters with differing prognoses and immune activation pathways. Cluster A tumors exhibited higher infiltration of both immune-activating cells (CD8^+^ T cells, NK cells, Th cells) and immune-suppressive cells (Macrophages, Treg cells) compared to Cluster B. High immune cell infiltration correlated with better prognosis and indicated a favorable response to immunotherapy. Experimental validation of PDCD1LG2, IGJ, and CSF2RB supported the expression trends of the bioinformatics findings.

Immune cell recruitment plays a fundamental mechanistic role in shaping the tumor microenvironment (TME) and influencing cancer progression and patient prognosis. The TAM population bifurcates into immunostimulatory M1 and tumor-promoting M2 phenotypes, the latter achieving immune evasion through cytokine-mediated suppression [26]. Regulatory T cells (Treg) suppress immunity through inhibitory cytokines and surface molecules. Increased intratumoral Treg levels correlate with poorer survival outcomes [27,28]. Tumor-infiltrating neutrophils play context-dependent roles in oncogenesis, with N1-polarized subsets exerting anti-tumor effects while N2-polarized populations facilitate tumor progression [29]. CD8^+^ T cell-mediated tumor cytotoxicity involves interferon-γ (IFN-γ) production [30]. Natural killer (NK) cells are innate cytotoxic lymphocytes capable of eliminating virus-infected and malignant cells. NK cell-based therapies have emerged as a pivotal frontier in cancer immunotherapy innovation [31]. Helper T cells, namely Th cells, can be categorized into two subsets: Thl and Th2. Thl cells mainly secrete cytokines such as IFN-γ and interleukin (IL)-2 and IL-12, which can activate macrophages. Th2 lymphocytes predominantly produce immunoregulatory cytokines including IL-4, IL-5, IL-10 and so on, which play a role in the immune response against viral infection and intracellular bacterial infection [32]. In this study, immune cell infiltration was enriched in Cluster A, counteracting the development of TNBC and leading to better tumor prognosis. It is hypothesized that infiltrating immune cells might be the main contributors to antitumor activity, making TNBC patients with certain infiltrating immune cell characteristics more sensitive to immunotherapy and achieving better treatment outcomes [33].

Immune checkpoints are paired receptor ligand molecules that inhibit immune response. These immune checkpoints participate in immune activation and can function as a natural inhibitory feedback loop to reduce inflammation, prevent normal tissue involvement, and act as central orchestrators of immunological balance [34]. The first immune checkpoint receptor discovered and identified was CTLA-4, which was the first to be associated with cancer treatment. In animal experiments, blocking CTLA-4 antibody can mediate tumor regression [35]. In addition to CTLA-4 inhibitors, programmed death protein 1 (PD-1)/programmed death ligand 1 (PD-L1) ICIs have also been approved and widely used by FDA. PD-1/PD-L1 axis can regulate physiological immune homeostasis, down-regulate inflammatory response, and may promote immune escape of cancer cells [36,37]. Blocking PD-1/PD-L1 can enhance the overall survival rate of many types of cancer and significantly improve the treatment response rate [38]. LAG3 can be expressed in lymphocytes, including CD4^+^ T cells, CD8^+^ T cells, NK cells and Treg cells [39]. The expression of LAG3 functionally interconnect with that of other immune checkpoints. In BC, the co-expression of LAG3, PD1/PD-L1 and TIGIT has been confirmed [40]. In our study, high expression of CD274, CTLA4, LAG3, PDCD1 and TIGIT suggested that the immunotherapy effect was meaningful.

Cross-cancer validation and its interpretation. In this study, we performed cross-cancer validation using immunotherapy cohorts from lung cancer (GSE135222) and melanoma (GSE91061). We acknowledge that these cancer types differ substantially from TNBC in terms of genetic background, tumor microenvironment, and clinical features. Therefore, the results from these cohorts should be interpreted as supportive evidence for the generalizability of our lactylation-related gene signature, rather than direct proof of its predictive value specifically in TNBC. The rationale for including these cohorts is twofold. First, the core mechanisms of immunotherapy response, including T cell activation, antigen presentation, and interferon signaling, are shared across multiple cancer types. Second, if our signature can effectively distinguish responders from non-responders in genetically and immunologically distinct tumor types, it would strengthen the confidence that this signature captures fundamental immune response-related biological features, rather than being merely a TNBC-specific artifact. Nevertheless, prospective studies with dedicated TNBC immunotherapy cohorts are urgently needed to validate the clinical utility of our model.

Ultimately, the experimental validation of the 3 hub genes (PDL2, IGJ, and CSF2RB) were consistent with previous research findings. Programmed Cell Death 1 Ligand 2 (PDCD1LG2), a member of the B7 family and a ligand for PD-1 alongside PDL1, has a higher affinity than PDL1, allowing it to synergistically modulate immune responses [41]. High PDL2 expression has been linked to BC progression, promoting cell growth, invasion and migration [42]. Despite the association of PDL2 expression with adverse clinical and pathological features, it may still serve as a positive prognostic marker for TNBC due to its potential to elicit robust anti-tumor immune responses [43]. The IGJ gene is expressed in various cells, including dendritic and mammary epithelial cells [44], and its levels vary under different pathological conditions. As a marker of BC, IGJ shows high accuracy in distinguishing BC tissue from normal tissue [45,46,47,48], though its role in malignant tumors remains underexplored. Colony-stimulating factor 2 receptor beta (CSF2RB) is involved in several cellular processes, including survival and differentiation [49]. RUNX1 has been shown to bind to the CSF2RB promoter, enhancing its transcription and reducing cell survival and metastasis [50]. A potentially oncogenic CSF2RB mutation (S230I) in BC patients has been identified, which may help develop new treatments [51].

It is worth noting that the expression levels of PDCD1LG2, IGJ, and CSF2RB differed not only between TNBC cells and normal breast cells (MCF-10A) but also between the two TNBC cell lines, BT-549 and MDA-MB-231. These two cell lines represent different molecular subtypes of TNBC: MDA-MB-231 is typically classified as a mesenchymal-like (basal B) subtype, while BT-549 exhibits characteristics of the mesenchymal stem-like subtype. Therefore, the observed inter-cell-line differences may reflect intrinsic biological heterogeneity among TNBC subtypes rather than experimental variation. Future studies using a broader panel of TNBC cell lines representing all molecular subtypes are needed to further validate the generalizability of our findings.

Certainly, this work acknowledges inherent research limitations. Primarily, the transcriptomic data sourced from public repositories exhibit certain sample size constraints. This inadequacy may cause individual differences among samples to account for a significant proportion in the analysis results, thereby affecting the general applicability of the conclusions drawn. Furthermore, although qRT-PCR, WB, and Confocal IF Microscopy techniques were employed to confirm the transcriptional levels of identified hub genes, further mechanistic studies are warranted to elucidate the tumor-specific functions of these hub genes in TNBC pathogenesis. However, these experiments primarily confirm expression differences and do not establish the functional roles or mechanisms of these genes in TNBC. In particular, claims regarding the potential of these genes as therapeutic targets or their involvement in immunotherapy response remain speculative at this stage. It is urgent to conduct more in-depth *in vivo* and *in vitro* experiments for further verification and exploration.

To summarize, we analyzed the lactylation-related genes of TNBC through database and divided them into 2 clusters by R language. We identified the DEGs of TNBC and conducted functional enrichment analysis of these DEGs, as well as predictions of immunotherapy efficacy and survival prognosis. Through *in vitro* analyses, we confirmed mRNA and protein expression patterns of three candidate hub genes (PDCD1LG2, IGJ, CSF2RB), corroborating prior bioinformatic predictions. These findings advance understanding of TNBC molecular mechanisms and provide a foundation for future studies to explore the therapeutic potential of these genes in TNBC.

## Data Availability

The data that support the findings of this study are available from the Corresponding Author, Rui Yang, upon reasonable request.

## Supplementary Materials

The supplementary material is available online at https://www.techscience.com/doi/10.32604/or.2026.078051/s1.

## Abbreviations

BC	breast cancer
CI	confidence interval
CNV	copy number variation
CSF2RB	Colony-stimulating factor 2 receptor beta
DEGs	differentially expressed genes
ER	estrogen receptor
FGES	Functional gene expression signatures
GEO	Gene Expression Omnibus
GO	Gene Ontology
GSVA	Gene set variation analysis
HER2	human epidermal growth factor receptor 2
HR	hazard ratio
IF	immunofluorescence
IFN-γ	interferon-γ
IL	interleukin
IPS	Immune cell Proportion Score
KEGG	Kyoto Encyclopedia of Genes and Genomes
K-M	Kaplan-Meier
NK	Natural killer
PCA	principal component analysis
PD-1	programmed death protein 1
PDCD1LG2	Programmed Cell Death 1 Ligand 2
PD-L1	programmed death ligand 1
PR	progesterone receptor
qRT-PCR	quantitative real-time polymerase chain reaction
ssGSEA	single sample Gene Set Enrichment Analysis
SNV	single nucleotide variation
TAMs	tumor associated macrophages
TCGA	The Cancer Genome Atlas
TME	tumor microenvironment
TNBC	triple-negative breast cancer
Treg	Regulatory T cells
WB	Western blot
