# Supplementary material for: Prognosis and Immunotherapy Effect of Triple-Negative Breast Cancer by Lactylation-Related Genes and Experimental Validation
Source: Oncol Res. 2026 Jun 16;34(7):29. doi: 10.32604/or.2026.078051 (PMC13292059; doi:10.32604/or.2026.078051)
Supplement: Supplementary file 1 [file OncolRes-34-78051-s001.zip › Supplementary_Figures.pdf]

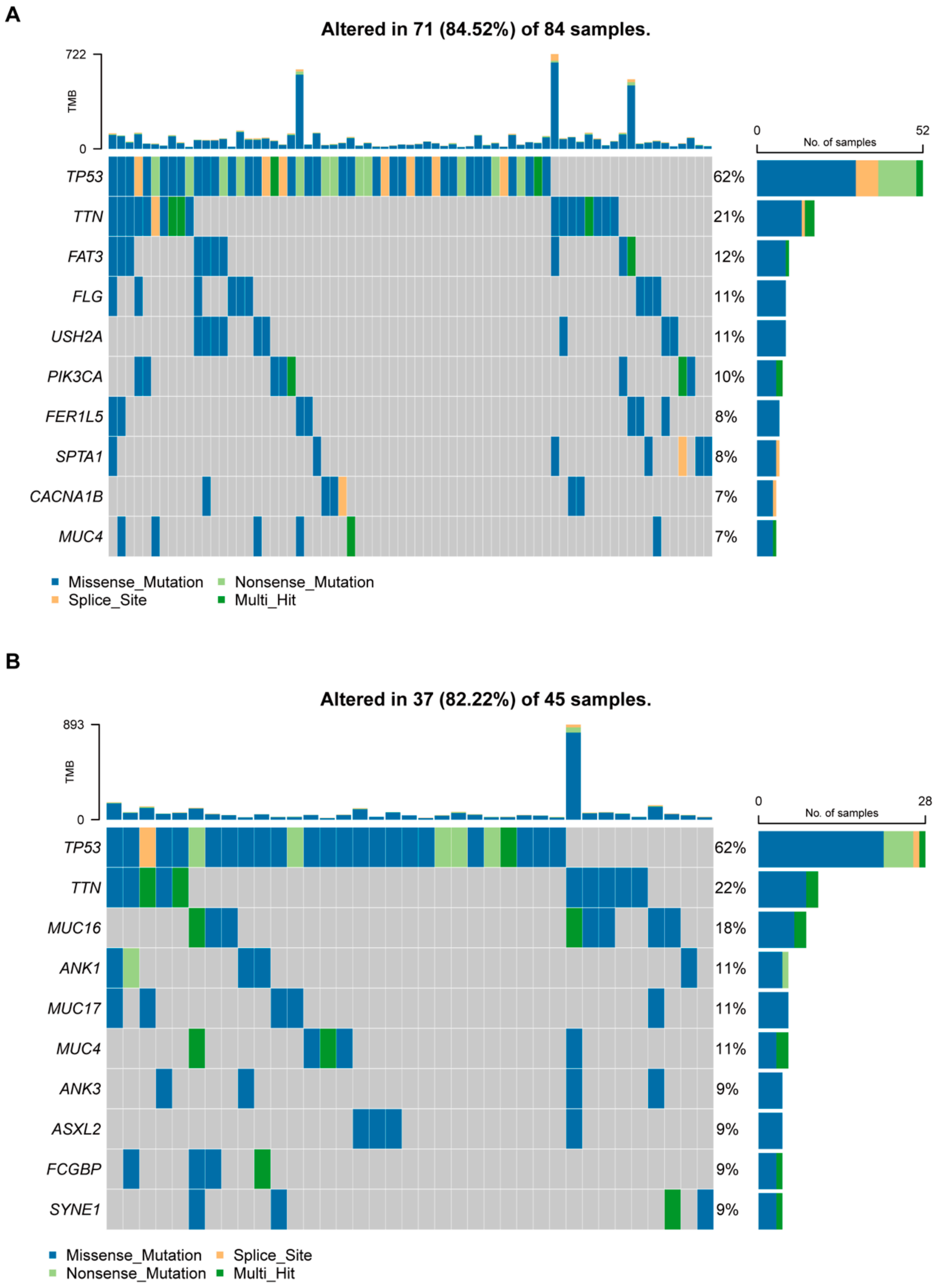

Supplementary Fig. S1 Status of gene mutations. **(A)** Waterfall map of somatic mutations in the high-score group. **(B)** Waterfall map of somatic mutations in the low-score group.

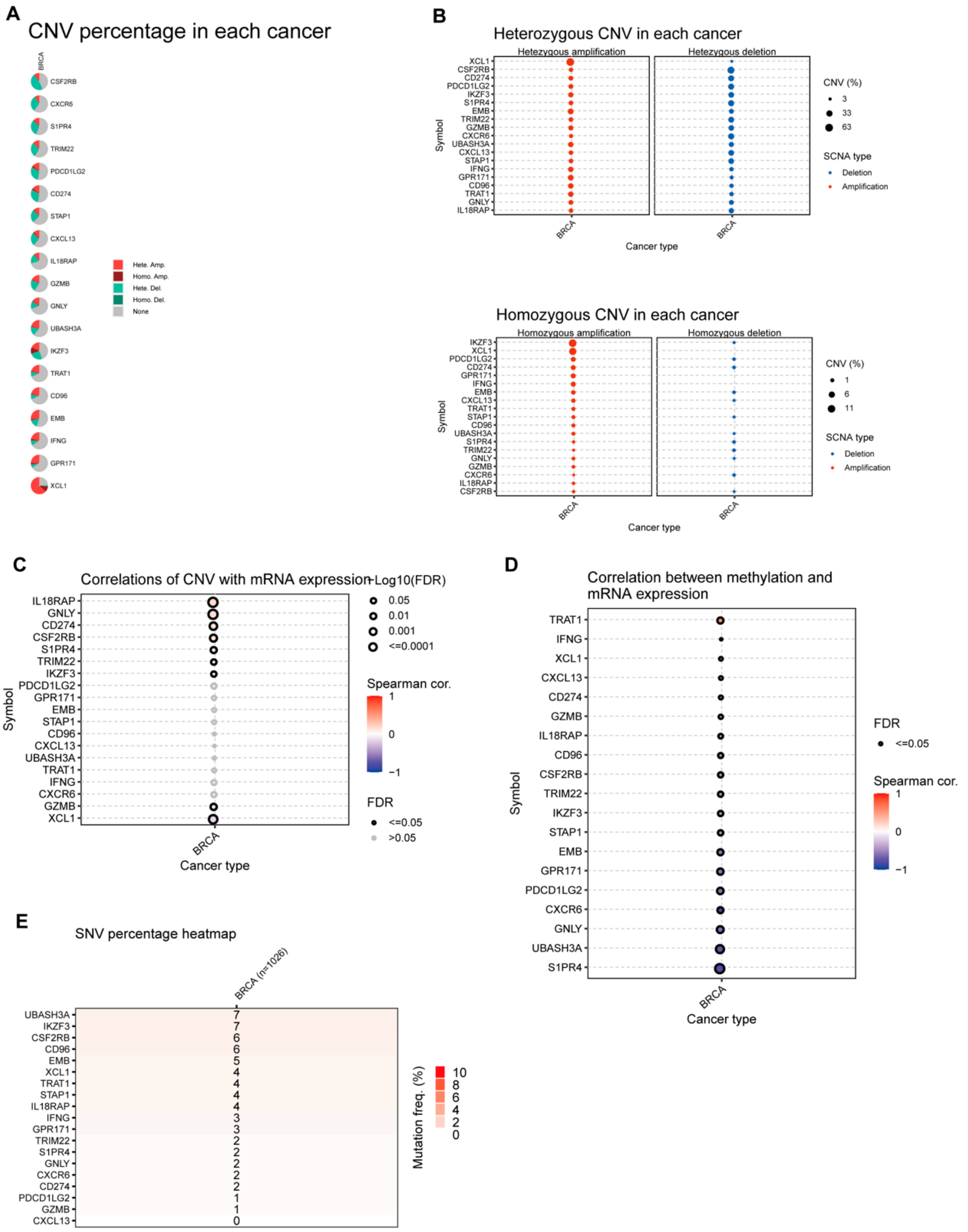

Supplementary Fig. S2 Genomic and Epigenetic Landscape. **(A)** CNV distribution in BRCA (pie chart). **(B)** Percentage of heterozygous or homozygous CNV. **(C)** Correlation between CNV and mRNA expression. **(D)** Correlation between methylation and mRNA expression. **(E)** Deleterious mutation frequency in BRCA.
